# Effects of E-Learning Environment Use on Visual Function of Elementary and Middle School Students: A Two-Year Assessment—Experience from China

**DOI:** 10.3390/ijerph17051560

**Published:** 2020-02-28

**Authors:** Zhixin Zhang, Gang Xu, Jing Gao, Lu Wang, Yonghai Zhu, Zhiyong Li, Wei Zhou

**Affiliations:** 1 College of Education, Capital Normal University, Beijing 100048, China; zhangzhixin@cnu.edu.cn (Z.Z.); 2167502012@cnu.edu.cn (J.G.); 3754@cnu.edu.cn (L.W.); 2 School of Sport Science, Beijing Sport University, Beijing 100084, China; 0738@bsu.edu.cn; 3 Elementary Education College, Capital Normal University, Beijing 100037, China; 4 Education Department, Huainan Normal University, Huainan 232001, China; zyli@hnnu.edu.cn; 5 Institute of Psychology, Capital Normal University, Beijing 100048, China; zhouwei@cnu.edu.cn

**Keywords:** E-learning environment and health, elementary and middle school students, visual function, vision, assessment

## Abstract

This two-year follow-up assessment was performed on 721 elementary (Grades 2–4) and middle (Grade 1) school students who used, and 62 Grade 4 (Control) students who did not use, E-learning environments from schools in Beijing and Shandong Province, China. Statistical analysis included repeated-measures single-factor and two-factor analyses of variance, and analysis of covariance (ANCOVA). In three assessments over two years, the students’ visual acuity, visual field, depth perception, and horary visual acuity were monitored, along with the related differences and developmental changes and the effect of the E-learning environment on these indexes: (1) For the first time, the average values of four indexes of visual function of the students exposure to the E-learning environment were obtained, among which the ratio of poor visual acuity was still high; (2) visual acuity and depth perception in middle school students was poorer than that of elementary school students, but their visual field and horary visual acuity was higher; (3) for the two years, the four indexes of the visual function of students in different grades showed different change trends; and (4) the comparison for G4 and control demonstrated that the frequency of E-learning environment use (6.75 h/week for G4) had no significant effect on the visual acuity and depth perception of the Grades 4 and 5 students in elementary school but had a significant effect on their visual field and horary visual acuity. However, in all of the included students, the E-learning environment use time significantly affected the left and right eye visual acuity in the students, except in G4.

## 1. Introduction

With the development of information technology, the use of an E-learning environment has become prevalent worldwide; moreover, it has also become part of the daily lives of elementary and middle school students, which has increased concerns regarding its effect on students’ health among their parents and schoolteachers, as well as the general public. Since 1985, studies conducted in China on the physical health of young students have demonstrated that the myopia rate of students has been high; moreover, the ratio of poor visual acuity detected has also demonstrated a continuous increasing tendency, and the age at which students were found to be myopic has gotten younger and younger [1]. This is likely a consequence of the increased use of E-learning environments by young children. On 30 August 2018, eight departments of the Chinese government, including the Ministry of Education and National Health Commission, jointly resolved to implement the Comprehensive Plan to Prevent Nearsightedness Among Children and Teenagers, with the goal of reducing myopia’s prevalence among children and adolescents in China to approximately 3% by 2030.

In addition to China, myopia’s prevalence is high among children in Southeast Asian countries, whereas it is low among adolescents in West Asian, African, Oceanian, and American countries [2]. The prevalence of myopia and high myopia varies according to region and ethnic group. On the basis of ethnic, economic, environmental, and other data, the global incidence of juvenile myopia and high myopia is expected to significantly increase during 2000–2050 [3]. Moreover, some scholars have indicated that myopia prevalence depends on genetic factors (e.g., ethnicity); nevertheless, environmental factors (e.g., decrease in outdoor activities) also influence myopia development. For instance, among children, increased educational pressure and lifestyle changes have reduced the time spent on outdoor activities—this can later result in myopia development [4]. Although several genes are related to high myopia, the question has thus far been discussed mainly from the perspectives of myopia prevalence, ethnic differences, and outdoor sports activities.

This study attempts to carry out empirical research from several dimensions of visual function and E-learning environment. The results of the empirical study may lay the foundation for the relevant decision-making of governments, schools, medical and health institutions, families, students, and other entities to care for children’s eyes and solve health problems (e.g., vision decline) during the digital education reform process in Southeast Asian countries.

## 2. Literature Review

Before the year 2000, the use of E-learning environments was not common in China and the world. At that time, researchers found that the main factors contributing to myopia in adolescents were heavy schoolwork load, long-term reading and writing at close range, neglect of physical exercise, poor lighting condition in the learning environment, and genetics [5,6]. Some empirical studies show that the Chinese government improved the learning conditions of students in various schools, especially the lighting conditions in classrooms, which had a certain effect on controlling the rate of myopia [7].

After entering the 21st century, the E-learning environment is becoming more and more popular in the world, and relevant research is also increasing. Computer use can affect vision: According to the American Academy of Ophthalmology (AOA), extensive computer use can lead to eye fatigue, redness, blurred vision, myopia, and other eye symptoms [8]. Kozeis found that viewing computer screens regularly can lead to eye discomfort, blurred vision, fatigue, headaches, and other symptoms [9]. Taptagaporn reported that in the process of using a computer, the most common symptoms are burning eyes and muscle pain, which are related to computer use duration [10]. In China, studies have indicated that the burden of classwork has some negative impacts on students’ vision. Yang et al. asserted that homework remains the main factor in vision decline among students [11]. Yu et al. studied the impact of using an online teaching environment in the classroom on students’ vision and found that computer use in class was not the main reason for the decline in students’ vision. The authors reported that students’ vision can be improved by improving the efficiency of classroom teaching and reducing the burden of schoolwork [12]. Zheng et al. studied the history of myopia prevention, treatment, and visual protection development worldwide; the authors reported that, among students, myopia is caused by the excessive growth of the visual axis, particularly that of visual acuity, which is caused by long-term reading and writing at close range, the heavy burden of schoolwork and homework, excessive pressure, high anxiety, lack of sleep and exercise, and nutritional imbalance [13,14].

Systematic and large-scale empirical studies on the effects of the E-learning environment on the visual function of elementary and middle school students and their scientific implications are lacking globally. Therefore, the Ministry of Education of China has set up a special project in which Capital Normal University, in collaboration with the General Administration of Sport, Beijing Sport University, and other relevant scientific research institutions, performed a large-scale two-year assessment.

Studies on visual function in students exposed to the E-learning environment have mostly been limited to assessing vision, which reflects spatial visual acuity and central vision in visual function but is not equivalent to visual function. Visual function also includes the peripheral vision, depth perception, and horary visual acuity, all of which are equally important in the development of elementary and middle school students. Therefore, the current study assessed not only the changes in elementary and middle school students’ vision, but also their visual field, visual depth (i.e., index of depth perception ability), and flicker fusion frequency (i.e., index of horary visual acuity).

E-learning environments include network teaching environments, as well as mobile learning environments. In elementary and middle schools, E-learning environment teaching equipment includes interactive electronic whiteboards or touch all-in-one machines, tablet computers, and Internet notebooks. In this study, the use time of the E-learning environment is the sum of the in- and after-class use time of the aforementioned equipment, specifically grouped as the in- and after-class use time of (1) electronic whiteboards or touch all-in-one machines; (2) all types of tablet computers and Internet notebooks; and (3) tablet computers or Internet notebooks, excluding smartphones and televisions.

Visual acuity refers to the maximum ability to distinguish the shape, size, and fine structure of an object with the eyes [15]. Zheng et al. reported that children’s vision can reach the level of adults’ vision at the age of six years, and thus, the most important period for the prevention of myopia is 3–6 years of age. Moreover, myopia prevention should last at least until the end of university education [16]. Logarithmic visual acuity charts are standard tools for visual acuity assessment and are thus widely used in optometric research and clinical applications [17]. The Eye Optometry Group of the Ophthalmology Branch of the Chinese Medical Association and the Eye Optometry Professional Committee of the Ophthalmologists Branch of the Chinese Medical Association also recommended the use of the standard logarithmic visual acuity chart in the Consensus of Experts on the Standardization of Testing Equipment and Setting Up in the Survey of Myopia Among Children and Adolescents (2019) [18].

Myopia refers to the visual distortion caused by the focus of the parallel light from 5 m away from the refraction system of the eye falling in front of the retina in the state of adjustment in the static state [19]. Diopters greater than −6.00D are defined as high myopia [20].

The visual field (i.e., peripheral vision) refers to all external scope and spatial factors of the visual angle noted when eyes are fixed in one position [21]. It strongly influences people’s actions, and even survival. According to the World Health Organization, a surrounding visual field of <10° represents a loss of peripheral vision, even when central vision is normal; this narrow visual field can affect daily life and work to some extent [22]. Visual field examination results can indicate the whole photographic function of the retinas and aid in evaluating the visual pathway and central vision function. They are also very helpful for the early detection of juvenile myopia and visual field damage from glaucoma [23]. Zhou asserted that visual field development is similar to that of other physiological indexes of the human body and increases with age; before the age of 10 years, visual field development is faster in female individuals than in male individuals, but this trend reverses after the age of 10 years. Nevertheless, visual field development in female individuals is complete 1–2 years earlier than in male individuals [22]. Perimeter (e.g., BD-II-108, Shanghai Yuanzhi Electronic Technology Co., Ltd., Shanghai, China) is a professional ophthalmic instrument used for measuring the visual field of the eyeball; it can provide detailed evaluation results for the upper, lower, inner, and outer directions of both the left and right eyes. After measurement, the tester only needs to connect the four points on the visual field map to obtain the range of the white visual field [22,24].

Depth perception refers to the perception of distance or depth of an object; it is realized by the cooperative activities of perception, such as seeing, listening, and moving, for which depth perception plays a leading role [25]. Depth perception ability is used as a crucial selection standard in football, basketball, and other sports [25,26]. Ji indicated that depth perception development involves both natural and training growth factors; of these, training growth factors are the main promoter of depth perception development [27]. In total, 73%–74% of 4-year-old children present stereovision, whereas approximately 20% demonstrate delayed development, with approximately 14% not demonstrating stereovision even after 8 years of age. Moreover, with increasing age, eye regulation ability decreases, and stereopsis worsens gradually [28]. However, Chu reported that, at the ages of 14–22 years, depth perception is not affected by age factors [26]. A depth perception tester (e.g., EP503A, Shanghai East China Normal University Science & Educational Instrument Co., Ltd., Shanghai, China) is an instrument for studying visual acuity in depth. It can test the minimum visual error of distance or depth of both eyes and can be widely used in the examination or selection of vehicle drivers, athletes, and other personnel required to have good depth perception, as well as in psychological experiments [29].

Horary visual acuity refers to the eyes’ ability to distinguish the time characteristics of movement changes in things, which makes it an important indicator to judge the level of vision. Horary visual acuity is typically expressed by the maximum fusion frequency of a flash that human eyes can grasp. The higher the flicker fusion frequency, the higher the horary visual acuity [30]. This index can be used in the early diagnosis of glaucoma [31]. It is also commonly used for determining mental fatigue [32]. Yu et al. reported that the flicker fusion frequency is linearly and negatively correlated with age—specifically, it decreases with age [33]. However, a study on flight fatigue in international flight pilots demonstrated no significant correlation between flicker fusion frequency and age factors [34]. A luminescent spot scintillator (e.g., EP403 Shanghai East China Normal University Science & Educational Instrument Co., Ltd., Shanghai, China) is an experimental instrument designed according to the principle of fusion critical frequency that can directly measure the critical frequency [35,36].

In general, this study analyzed the current situation concerning the visual function of elementary and middle school students in China by testing and evaluating the core indicators of visual function. The aim was to provide an empirical basis on which the government can formulate visual health standards for elementary and middle school students and a manual for the E-learning environment, and provide pertinent information to parents, schools, and the community on how to protect the vision and visual function of elementary and middle school students.

## 3. Methodology

### 3.1. Participants

In September 2014, 21 test classes (721 students; experience of using an E-learning environment = 0–1 year) and 1 control (Grade 4; denoted as Control) class (*n* = 62 students, E-learning environment experience = 0 years. The vast majority of schools in China have considered equal access to E-learning equipment between different classes in the same grade, which is the main reason for the lack of control classes) were selected from Beijing (*n* = 242) and Shandong Province (*n* = 541) in China. Of the 721 test students, 107, 176, and 254 were in Grades 2, 3, and 4 of elementary school, respectively (hereafter represented as G2, G3, and G4, respectively), and 184 students were in Grade 1 of middle school (hereafter represented as G7). The average age of all students was 10.02 years, with 402 boys and 381 girls included. Over a 2-year follow-up, three large-scale assessments were conducted on all enrolled students.

Informed consent was obtained from all students’ school administration and their parents. The specific procedures and ethics of this study were reviewed and approved by the Ministry of Education of China, the competent department of the project. The approval date of the project was 20 March 2014, and the project number is MCM20130602.

### 3.2. Assessment Tools

Standardized logarithmic visual acuity charts, a BD-II-108 perimeter, the EP503A depth perception tester, and an EP403 luminescent spot scintillator were used separately to evaluate visual acuity, visual field, depth perception, and horary visual acuity in the sample students.

### 3.3. Assessment Process

First (baseline) assessment (Assessment 1) data were obtained during September and October 2014; thereafter, second assessment (Assessment 2) and third assessment (Assessment 3) data were obtained during September and October 2015 and September and October 2016, respectively. Some elementary schools in Shandong Province follow a 5-year system, so Assessment 3 of these primary schools was advanced to the spring semester of Grade 5 (June 2016).

### 3.4. Data Processing and Analysis

First, study data were inputted into and organized using MS Excel 2016 and then processed and analyzed on SPSS (version 19.0). Correlation-sample single-factor analysis of variance (ANOVA; repeated quantity) was used to explore the visual acuity, visual field, depth perception, and horary visual acuity of the sample students over the two study years, whereas independent-sample single-factor ANOVA was used to explore visual acuity differences in each grade in all three assessments. Next, the independent-sample *t*-test was used to explore any differences in the indicators between the test and control classes, and two-factor mixed-design ANOVA was employed to explore the interaction of higher-grade class data with that of lower-grade classes and of short-term use data with that of long-term use.

## 4. Results

### 4.1. Visual Acuity: Status, Difference, and Development

#### 4.1.1. Overall Visual Acuity

The visual acuity data of the assessment were calculated according to the national standards in China and students’ physical health evaluation results. Visual acuity is normal if the distance vision of both eyes is ≥ 5.0 and poor if it is < 5.0. Here, if the visual acuity of both eyes was inconsistent, the vision of the lower eye prevailed. According to the three assessments, the average low visual acuity prevalence in the elementary and middle school students was 45.9% and 71.0%, respectively; these values were similar to and lower than those for elementary (45.7%) and middle (74.4%) school students in the 2014 China Student Physique and Health Research Report, published by China’s Ministry of Education, General Administration of Sport, and other relevant departments.

#### 4.1.2. Visual Acuity Differences in Students of Different Grades at Different Assessment Timepoints

**Figure 1:**
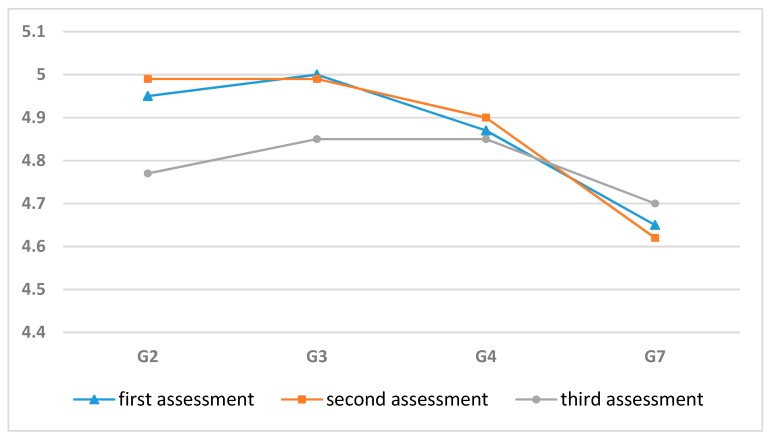
Horizontal comparison of the visual acuity of students in different grades.

As shown in Figure 1, by using independent-sample single-factor ANOVA, the students’ visual acuity data in the three assessments were compared horizontally for each grade:

(1) Assessment 1:

All classes demonstrated significant between-group differences in visual acuity (F (3, 729) = 15.692, *p* < 0.05). Moreover, compared with G7, G2, G3, and G4 demonstrated significant differences (*p* < 0.05). Left eye visual acuity differed significantly between G3 and G4 (*p* < 0.05). Finally, visual acuity was lower in G7 than in G4, lower in G4 than in G3 and G2, and similar in G3 and G2;

(2) Assessment 2 (two semesters (one year) after Assessment 1):

G4 demonstrated significant differences in visual acuity among students (F (3, 688) = 18.752, *p* < 0.05). Similar significant between-group differences were noted for the visual acuity of students in G2, G3, and G4 of elementary school and G7, respectively (*p* < 0.05). Finally, visual acuity was lower in G7 than in G2, G3, and G4, and similar in G2, G3, and G4;

(3) Assessment 3 (3–4 semesters (1.5–2 years) after Assessment 1):

All four classes demonstrated significant between-group differences in right eye visual acuity (F (3, 489) = 3.942, *p* < 0.05) but not in left eye visual acuity (F (3, 500) = 2.593, *p* > 0.05). Moreover, the in-group differences in right eye visual acuity were significant in G3, G4, and G7 (*p* < 0.05). In general, with E-learning environment use, visual acuity was lower in G7 than in G3 and G4, whereas results in G2, G3, and G4 were similar.

In summary, after exposure to an E-learning environment, the higher the grade, the worse the students’ visual acuity.

#### 4.1.3. Developmental Visual Acuity Changes in Students of the Same Grade at Different Assessment Timepoints

(1) Changes in visual acuity in G2 over Grades 2–4 movement in elementary school:

In G2, the ratio of poor visual acuity was 37.62% at Assessment 1 (Grade 2), increased to 40.63% at Assessment 2 (Grade 3, but with very high F- and *p*-values), and, finally, to 49.38% at Assessment 3 (Grade 4), thus indicating a worsening of vision with age after exposure to the E-learning environment.

Table 1 presents the result of the single-factor ANOVA (number of repetition) employed to explore changes in the visual acuity in G2 students over the three assessments (F (1.454, 123.601) = 47.322, *p* < 0.01); thus, left eye visual acuity differed over the assessment timepoints. The average left eye visual acuity in the three assessments reached a significant level (*p* < 0.05), demonstrating an increasing trend first, followed by a decreasing trend. Thus, over the movement from Grade 2 to Grade 4 in elementary school, left eye visual acuity in G2 first significantly improved and then significantly worsened. Moreover, G2 students exhibited significantly lower left eye visual acuity in Grade 4 than in Grade 2. This result was similar for right eye visual acuity.

In summary, over the Grades 2–4 movement in elementary school, the visual acuity of both eyes changed significantly in G2 by first improving and then worsening, leading to their visual acuity becoming significantly lower in Grade 4 than in Grade 2.

(2) Changes in visual acuity in G3 over the Grades 3–5 movement in elementary 5 school:

In G3, the ratio of poor visual acuity was 36.93% at Assessment 1 (Grade 3), increased to 37.65% at Assessment 2 (Grade 4), and, finally, to 50.00% at Assessment 3 (Grade 5), thus indicating a worsening of vision with age after exposure to the E-learning environment.

The results of a single-factor ANOVA demonstrated significant differences in the average left eye visual acuity over the three assessments in G3 (intergroup effect: F (1.719, 192.546) = 27.073, *p* < 0.01). Assessments 1 and 2 demonstrated stable left eye visual acuity values, but Assessment 3 did not: It remained stable at first and then decreased significantly. This result was similar for right eye visual acuity.

In summary, over the Grades 3–5 movement in elementary school, the visual acuity of both eyes changed significantly in G3 by first remaining stable and then worsening;

(3) Changes in visual acuity in G4 over the Grades 4–6 movement in elementary school:

In G4, the ratio of poor visual acuity was 50.70% at Assessment 1 (Grade 4) and decreased to 46.21% at Assessment 2 (Grade 5) but finally increased to 60.17% at Assessment 3 (Grade 6), thus indicating a worsening of vision with age after exposure to the E-learning environment, particularly during the Grades 5–6 transition period.

Correlation-sample single-factor ANOVA (repeated quantity) demonstrated no significant differences in average left eye visual acuity among the three assessments in G4 (between-group effect: F (2, 222) = 2.113, *p* > 0.05). This result was similar for right eye visual acuity.

In summary, over the Grades 4–6 movement in elementary school, G4 demonstrated no significant changes in visual acuity. Notably, poor visual acuity was sensitive to change, but the differences in average visual acuity over time were not; however, the differences in the trend of change between the two did not demonstrate a considerable contradiction.

(4) Changes in visual acuity in G7 over the Grades 1–3 movement in middle school:

In G7, the ratio of poor visual acuity was 68.92% at Assessment 1 (Grade 1), increased to 71.76% at Assessment 2 (Grade 2; but with very high F and *p* values), and, finally, to 83.58% at Assessment 3 (Grade 3), thus indicating a considerable worsening of vision with age after exposure to the E-learning environment.

Correlation-sample single-factor ANOVA (repeated quantity) demonstrated significant differences in the average left eye visual acuity among the three assessments in G7 (between-group effect: F (1.728, 108.893) = 4.049, *p* < 0.05). The highest average left eye visual acuity was noted in Assessments 1 and 2. The average left eye visual acuity remained stable at first and then increased significantly. Therefore, over the Grades 1–3 movement, G7 demonstrated significant changes in visual acuity by remaining stable at first and then improving significantly: It was significantly higher in Grade 3 than in Grades 1 and 2. Regarding right eye visual acuity, G7 demonstrated significant differences in Assessments 2 and 3 but not Assessment 1: Their right eye visual acuity was significantly higher in Grade 3 than in Grade 2.

In summary, over the Grades 1–3 movement in middle school, the visual acuity of both eyes remained stable at first and then improved significantly.

### 4.2. Visual Field: Status, Difference, and Development

#### 4.2.1. Overall Visual Field

The average upper, lower, inner, and outer direction visual field values in all elementary school students (G2, G3, and G4) in the three assessments were, respectively, 44.55, 57.93, 54.86, and 66.03 for the left eye and 44.83, 58.31, 55.84, and 65.88 for the right eye; in G7, they were, respectively, 47.27, 66.13, 60.65, and 77.35 for the left eye and 48.17, 67.01, 60.20, and 73.57 for the right eye.

#### 4.2.2. Visual Field Differences in Students of Different Grades at Different Assessment Timepoints

**Figure 2:**
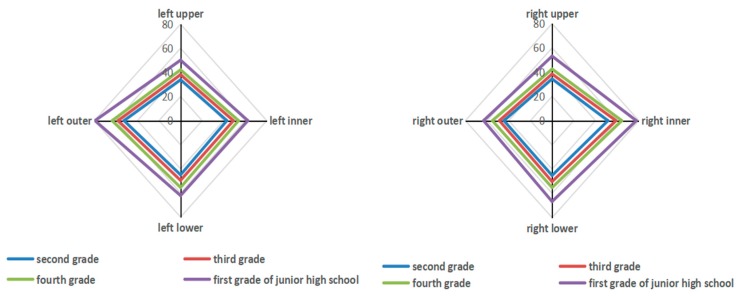
Horizontal comparison of students’ left and right eye visual fields in each grade at Assessment 1.

Figure 2 displays the representative average visual field value of students in different grades in Assessment 1. These data demonstrate that the visual field of students in higher grades was wider than that of students in lower grades, according with the general development of the adolescent visual field [22].

#### 4.2.3. Developmental Visual Field Changes in Students of the Same Grade at Different Assessment Timepoints

(1) Changes in the visual field in G2 over the Grades 2–4 movement in elementary school:

The correlation-sample single-factor ANOVA results demonstrate that differences in the average upper, lower, inner, and outer visual fields in the left and right eyes in G2 were significant (too many F- and *p*-values, so omitted). The upper left eye visual field differences were significant in Assessment 1 but not in Assessments 2 and 3. As shown in Table 2, the average of the three assessments demonstrated a gradually increasing trend. Moreover, for the internal orientation of the visual field in the left eye, there were significant differences in the values obtained in the three evaluations, and the average values also demonstrated a gradually increasing trend. For the general visual field, only Assessment 1 data reached a significant level: The average increased first and then decreased but without significance (leading to very high F- and *p*-values; decreased 0.2 and 2, respectively).

Next, for the right eye, the students’ visual field in the upper direction was significantly higher than that of the left eye in the upper direction, and the average value was gradually increased. Only Assessment 1 data for the internal orientation of visual field in the left eye were significant, with the average demonstrating a gradually increasing trend. For the lower and outer visual field, only Assessment 1 data were significant: The average increased first and then decreased, but without significance (leading to very high F- and *p*-values; decreased 0.1 and 0.7, respectively).

In summary, over the Grades 2–4 movement in elementary school, the visual field improved in G2.

(2) Changes in visual field in G3 over the Grades 3–5 movement in elementary school:

Correlation-sample single-factor ANOVA (repeated quantity) results demonstrate that the difference in the average upper, lower, inner, and outer visual field in G3 students was significant (very high F- and *p*-values). The visual field improved as G3 moved from Grade 3 to Grade 4. In Grade 5, however, the visual fields of the left and right eyes demonstrated a trend of variation; of these, the visual field out of the left eye (very high F- and *p*-values) was nonsignificant but that out of the right eye changed significantly.

(3) Changes in the visual field in G4 over the Grades 4–6 movement in elementary school:

Correlation-sample single-factor ANOVA results demonstrate that the difference in the average upper, lower, inner, and outer visual field in G4 students was significant (very high F- and *p*-values). The visual field of the left and right eyes in G4 students improved gradually, but the average right and left eye visual fields and outer visual fields increased at first and then decreased significantly. In summary, in G4, the visual field was better in Grade 5 than in Grade 6 and better in Grade 6 than in Grade 4.

(4) Changes in the visual field in G7 over the Grades 1–3 movement in middle school:

Correlation-sample single-factor ANOVA results indicate that, in all three assessments, the average differences in the upper, lower, inner, and outer visual field in G7 were significant (very high F- and *p*-values). The upper and lower left eye visual field values were nonsignificant only in Assessments 2 and 3; in other cases, the average upper left eye visual field values first decreased and then increased, and Assessment 3 values were lower than Assessment 1 values, whereas, for the lower left eye visual field, only Assessment 3 values increased first and then decreased and were higher than Assessment 1 values. The inner and outer left eye visual field was the same as the upper left eye visual field. Consequently, over the Grades 1–3 movement in middle school, the upper, inner, and outer visual field in the left eye worsened and the corresponding lower visual field improved.

The results of Assessments 2 and 3 in the upper right eye visual field were nonsignificant, but the other values were significant; the average values first decreased and then increased, but Assessment 3 values were lower than Assessment 1 values. In Assessments 1 and 2, lower right eye visual field values were nonsignificant, whereas other values were significant, demonstrating a gradual increasing trend. The internal orientation of the visual field in the right eye demonstrated a significant difference among the three assessments, with the average values demonstrating a downward trend. Only the data of Assessments 1 and 2 for the external orientation of the visual field in the right eye were nonsignificant, whereas the other data were significant, demonstrating a downward trend. In summary, over the Grades 1–3 movement in middle school, the upper, inner, and outer right eye visual field worsened and the lower right eye visual field improved.

### 4.3. Depth Perception: Status, Difference, and Development

#### 4.3.1. Overall Status of the Depth Perception of Test Students

The average visual depth (i.e., index of depth perception) of the elementary and middle school students was 1.37 and 1.65, respectively.

#### 4.3.2. Depth Perception Differences in Students of Different Grades at Different Assessment Timepoints

Table 3 presents the change in the average visual depth in G2, G3, G4, and G7 over 2 years—the larger the value is, the worse the depth perception.

By using independent-sample single-factor ANOVA, the visual depth of all students was compared horizontally: No significant difference was noted among all students in Assessments 1 (F (3, 573) = 0.939, *p* > 0.05) and 3 (F (3, 399) = 2.245, *p* > 0.05). In Assessment 2, the differences in G2, G3, G4, and G7 were significant (F (3, 541) = 14.074, *p* < 0.01); furthermore, only the visual depth in G4 and G7 was nonsignificant and, with movement to a higher grade, visual depth increased, indicating a decrease in depth perception.

#### 4.3.3. Depth Perception Changes in Students of the Same Grade at Different Assessment Timepoints

(1) Changes in depth perception in G2 over the Grades 2–4 movement in elementary school:

Correlation-sample single-factor ANOVA (repeated quantity) results demonstrated that the average values from the three assessments in G2 were significantly different (F (1.224, 102.819) = 6.709, *p* < 0.01). Furthermore, no significant difference was noted between Assessments 1 and 3, but the other values exhibited significance, demonstrating a decrease first and then an increase. Thus, over the Grades 2–4 movement in elementary school, the depth perception of students significantly improved first and then decreased significantly, but depth perception did not differ significantly in G2 between Grades 2 and 4.

(2) Changes in depth perception in G3 over the Grades 3–5 movement in elementary school:

Correlation-sample single-factor ANOVA (repeated quantity) results show that the average values from the three assessments in G3 differed significantly (F (1.180, 129.819) = 4.805, *p* < 0.05). The difference between Assessments 1 and 3 was nonsignificant but was significant between others, with the average values decreasing first and then increasing. Thus, over the Grades 3–5 movement in elementary school, depth perception first improved significantly and then decreased significantly in G3, but the depth perception differences between Grades 2 and 5 were nonsignificant.

(3) Changes in depth perception in G4 over the Grades 4–6 movement in elementary school:

Correlation-sample single-factor ANOVA (repeated quantity) results demonstrate that the average difference in the results of the three assessments in G4 were nonsignificant (F (1.819, 229.162) = 2.704, *p* > 0.05).

(4) Changes in depth perception in G7 over the Grades 1–3 movement in middle school:

Correlation-sample single-factor ANOVA (repeated quantity) results indicate that the average difference in the three assessment results in G7 was significant (F (1.276, 85.524) = 9.357, *p* < 0.01). Post-hoc comparison found that there were significant differences in the values obtained in the three evaluations; the average value first increased and then decreased. Therefore, over the Grades 1–3 movement in middle school, depth perception in G7 significantly worsened and then improved significantly; however, depth perception was significantly worse in Grade 3 than in Grade 1.

### 4.4. Horary Visual Acuity: Status, Difference, and Development

#### 4.4.1. Overall Situation of Test Class Students

The average flicker fusion frequency (i.e., index of horary visual acuity) for the left and right eyes was, respectively, 40.99 and 40.60 in all elementary school students, and 44.37 and 45.41 in middle school students.

#### 4.4.2. Horary Visual Acuity Differences in Students of Different Grades at Different Assessment Timepoints

Table 4 presents the changes in the average flicker fusion frequency of all students over 2 years: The larger the value, the better the horary visual acuity.

By using independent-samples single-factor ANOVA, a horizontal comparison of the flicker fusion frequencies in each grade was performed: In Assessment 1, the difference in flicker fusion frequencies between the left and right eyes of the students in G2, G3, G4, and G7 was significant (F (3, 677) = 162.531, *p* < 0.01; F (3, 677) = 174.580, *p* < 0.01). Flicker fusion frequencies were significantly higher in students in higher grades than in those in lower grades, indicating that the higher the grade, the higher the horary visual acuity of the students. In Assessment 2, the difference in the left eye flicker fusion frequencies of the students in the four grades was significant (F (3, 655) = 6.544, *p* < 0.01)). Moreover, the left eye flicker fusion frequency in G3, G7, G4, and G7 was significant, and the left eye flicker fusion frequency in G2 was greater than that in G4 and G5. Similarly, the difference in the right eye flicker fusion frequencies among all students was significant (F (3, 655) = 4.419, *p* < 0.01). The right eye flicker fusion frequency of the Grade 7 students was significantly higher than that of the Grade 4 students. In Assessment 3, the difference in the left eye flicker fusion frequencies of all students was significant (F (3, 469) = 5.779, *p* < 0.01). The left eye flicker fusion frequencies in G2, G4, and G7 were also significant: The left eye flicker fusion frequency was higher in G2 than in G4 and G7. The differences in the right eye flicker fusion frequencies in all four grades were nonsignificant (F (3, 469) = 2.271, *p* > 0.05).

#### 4.4.3. Horary Visual Acuity Changes in Students of the Same Grade at Different Assessment Timepoints

The horary visual acuity differences in students of different grades in the three assessments were evaluated using correlation-sample single-factor ANOVA (repeated quantity). The average difference in the left eye flicker fusion frequency in G2 from three assessments reached a significant level (F (1.451, 127.666) = 280.611, *p* < 0.01). Moreover, the differences between the two groups in the three assessments were significant, and the regularity of horary visual acuity in the right eye was the same as in the left eye. Therefore, over the Grades 2–4 movement in elementary school, the students’ horary visual acuity improved. The results were similar for G3 with respect to the movement from Grade 3 to Grade 5 in elementary school.

The differences in the left eye flicker fusion frequency in G4 were significant (F (2, 264) = 315.996, *p* < 0.01). Post-hoc comparison found that there were significant differences in the values obtained in the three evaluations. Therefore, when moving to Grade 6, the left eye horary visual acuity in G4 students improved significantly and then worsened significantly; moreover, their left eye horary visual acuity was significantly higher in Grade 6 than in Grade 4. The differences in right eye flicker fusion frequencies in G4 were significant (F (2, 264) = 503.529, *p* < 0.01). Moreover, the difference between Assessments 2 and 3 in G4 was nonsignificant, whereas it was significant in other situations, with the average values demonstrating an increase first and then a decrease. Thus, over the Grades 4–6 movement in elementary school, right eye horary visual acuity in G4 significantly improved first and then worsened, and was significantly higher in Grade 6 than in Grade 4.

In G7, the differences in left eye flicker fusion frequencies were significant (F (1.252, 86.401) = 5.588, *p* < 0.05). The difference was significant only between Assessments 1 and 2, with the average values increasing first and then decreasing. Thus, left eye horary visual acuity in G7 was significantly higher in Grade 2 than in Grade 1 in middle school, but it decreased slightly, but nonsignificantly, in Grade 3 in middle school. Moreover, the difference in the average right eye flicker fusion frequencies in G7 was significant (F (1.238, 85.405) = 27.962, *p* < 0.01). The difference between the values of Assessments 1 and 2 was nonsignificant, but the difference was significant in other cases. The average values also demonstrated an upward trend, indicating that right eye horary visual acuity gradually improved in G7 over the Grades 1–3 movement in middle school.

### 4.5. Comparison of Visual Functions between Test and Control Classes

To further explore whether E-learning environment use affects students’ visual function, an independent-sample *t*-test on the data of G4 and Control in an urban elementary school in Shandong Province was employed (the dimensions of comparison here are the same as that of Section 4.1, Section 4.2, Section 4.3 and Section 4.4, and the statistical method used is not complicated. Considering the limitation of the paper’s length and the convenience for readers to read, only a concise description was made here, important evidence and conclusions were provided, and redundant process data tables were omitted).

#### 4.5.1. Comparison of Visual Acuity between G4 and Control

Visual acuity did not differ significantly between G4 and Control (t1 (111.817) = 1.312, p1 = 0.192 > 0.05; t2 (94) = −0.340, p2 = 0.735 > 0.05; t3 (90) = 1.709, p3 = 0.091 > 0.05).

#### 4.5.2. Comparison of Visual Field between G4 and Control

In Assessment 1, visual fields of the left and right eyes differed significantly between G4 and Control (*p* < 0.05). Students in G4 have a broader visual field.

In Assessments 2, visual fields of the left and right eyes did not differ significantly between G4 and Control (*p* > 0.05).

In Assessment 3, the upper and inner visual field of the left eye and upper and outer visual field of the right eye did not differ significantly between G4 and Control (*p* > 0.05); however, visual fields of the lower and outer left eye and lower and inner right eye differed significantly between G4 and Control (*p* < 0.05), and G4 exhibited a wider visual field.

**Figure 3:**
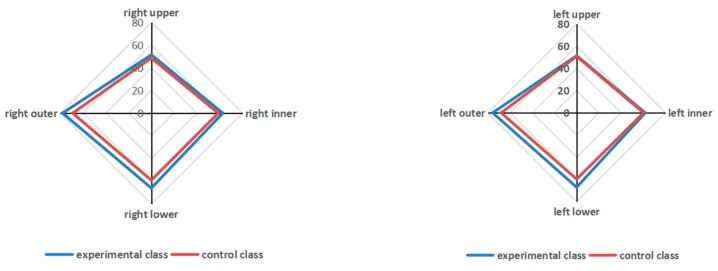
Comparison of visual fields in the left and right eyes between G4 and Control in Assessment 3.

In general, over a short-term period of nearly two semesters of E-learning environment use, the students’ visual field narrowed, whereas over a long-term period of three semesters (i.e., 1.5 years and longer), the students’ visual field shifted to the lower left (this may be related to the “phubbing phenomenon”; Figure 3).

#### 4.5.3. Comparison of Depth Perception between G4 and Control

Visual depth did not differ significantly between G4 and Control (t1 (114) = −0.381, p1 = 0.704 > 0.05; t2 (52.755) = 2.005, p2 = 0.050 < 0.05; t3 (103) = −0.014, p3 = 0.989 > 0.05).

#### 4.5.4. Comparison of Horary Visual Acuity between G4 and Control

Data from Assessments 1 and 2 did not differ significantly (*p* > 0.05). Moreover, Assessment 3 data were nonsignificant for the left eye (t (95) = −0.182, *p* = 0.856 > 0.05) but showed a significant difference for the right eye (t (95) = 2.779, *p* = 0.007 < 0.01). G4 had a higher flicker fusion frequency than the Control, indicating that visual acuity was improving in G4.

## 5. Discussion

### 5.1. ANOVA for the Interaction of Grade with Short- and Long-Term E-Learning Environment Use

To prevent false-positive errors and explore the interaction effect of the grade level with long- and short-term E-learning environment use, a two-factor mixed-design ANOVA was performed, and the results indicated that the main effect of the assessment timepoint on left eye visual acuity was nonsignificant (F = 1.763, *p* = 0.173). For left eye visual acuity, Mauchly’s W coefficient was 0.834 (c2 = 75.570, *p* < 0.01), indicating a violation of sphericity and thus confirming the correlation between the repeated assessment data. Greenhouse–Geiser correction results also demonstrate that the main effect of the assessment timepoint on left eye visual acuity was nonsignificant (F = 2.461, *p* = 0.095). Nevertheless, the main effect of grade on left eye visual acuity was significant (F = 32.858, *p* < 0.001): The left eye visual acuity of the students in higher grades was lower than that of the students in lower grades.

The interaction of grade with the left eye visual acuity assessment timepoint was significant (F = 22.762, *p* < 0.001; details in Table 5). The simple-effect test results demonstrate significant differences in left eye visual acuity among the three assessments (F = 47.265, *p* < 0.001) in lower grades: The visual acuity in Assessment 3 was significantly lower than that in Assessments 1 and 2 (both *p* < 0.001), but the differences in the visual acuity in Assessments 1 and 2 were nonsignificant (*p* = 0.099). By contrast, in higher grades, these differences were nonsignificant between all three assessments (F = 2.182, *p* = 0.114). It should be noted that the later the assessment timepoint was, the longer the students had continued to use E-learning environments.

The two-factor mixed-design ANOVA showed that the main effect of the assessment timepoint on right eye visual acuity was nonsignificant (F = 1.441, *p* = 0.238). Mauchly’s W coefficient was 0.867 (c2 = 58.382, *p* < 0.01), indicating a violation of sphericity and thus confirming the correlation between the repeat assessment data. Greenhouse–Geiser correction results demonstrate that the main effect of the assessment timepoint on right eye visual acuity was nonsignificant (F = 1.563, *p* = 0.212). However, the main effect of grade on right eye visual acuity was significant (F = 65.185, *p* < 0.001): The right eye visual acuity of the students in higher grades was lower than that of the students in lower grades.

The interaction of grade with the right eye visual acuity assessment timepoint was significant (F = 30.862, *p* < 0.001; details in Table 6). The simple-effect test results demonstrate a significant difference between right eye visual acuity in the three assessments (F = 50.829, *p* < 0.001) for the lower grades: Visual acuity in Assessment 3 was significantly lower than that in Assessments 1 and 2 (both *p* < 0.001), but the differences in visual acuity in Assessments 1 and 2 were nonsignificant (*p* = 0.142). In higher grades, these differences were significant between all three assessments (F = 5.919, *p* < 0.01): Visual acuity in Assessment 3 was significantly higher than that in Assessments 1 and 2 (*p* < 0.05), and visual acuity in Assessment 2 was significantly lower than that in Assessment 1 (*p* = 0.023).

Thus, the two-factor mixed-design ANOVA results indicate that the visual acuity of students in higher grades was lower than that of students in lower grades, and that the visual acuity of students in lower grades decreased as the assessment timepoints progressed (i.e., as the use of the E-learning environment was prolonged); whereas, in students in higher grades, it did not significantly change but improved.

The two-factor mixed-design ANOVA results demonstrate that the main effect of assessment timepoint on left eye flicker fusion frequency was also extremely significant (F = 160.570, *p* < 0.001). Mauchly’s W coefficient was 0.891 (c2 = 52.309, *p* < 0.01), indicating a violation of sphericity and thus confirming the correlation between the repeat assessment data. Greenhouse–Geiser correction also showed that the main effect of assessment timepoint on left eye flicker fusion frequency remained significant (F = 164.527, *p* < 0.001). A significant main effect of grade on left eye flicker fusion frequency (F = 68.889, *p* < 0.001) indicates that students in higher grades had a higher left eye flicker fusion frequency than lower grade students (i.e., horary visual acuity improved).

The interaction of grade with the assessment timepoint of left eye flicker fusion frequency was significant (F = 81.660, *p* < 0.001). The simple-effect test results revealed that in the lower grades, the differences in the left eye flicker fusion frequencies in the three assessments were significant (F = 724.176, *p* < 0.001), and the left eye flicker fusion frequency in Assessment 3 was significantly higher than in Assessments 1 and 2, whilst the left eye flicker fusion frequency in Assessment 2 was significantly higher than in Assessment 1 (*p* < 0.001); in the higher grades, the left eye flicker fusion frequencies in all three assessments differed significantly (F = 3.718, *p* < 0.01). Only Assessments 1 and 2 demonstrated a significant difference in the left eye flicker fusion frequency (*p* = 0.002), but the Assessment 2 value was significantly higher than the Assessment 1 value.

Two-factor mixed-design ANOVA results demonstrate that the main effect of the assessment timepoints of right eye flicker fusion frequency was highly significant (F = 145.229, *p* < 0.001). The Mauchly’s W coefficient was 0.254 (c2 = 619.932, *p* < 0.01), indicating a violation of sphericity and thus confirming the correlation between the repeat assessment data. Greenhouse–Geiser correction results showed that the main effect of the assessment timepoint of the right eye flicker fusion frequency remained highly significant (F = 52.858, *p* < 0.001). A significant main effect of grade on the right eye flicker fusion frequency (F = 44.190, *p* < 0.001) indicates that students in higher grades had higher right eye flicker fusion frequency than lower grade students (i.e., horary visual acuity became better).

The interaction of grade with the assessment timepoints of right eye flicker fusion frequency was significant (F = 15.364, *p* < 0.001). The simple-effect test results showed that, in lower grades, the right eye flicker fusion frequency of the three assessments differed significantly (F = 758.789, *p* < 0.001), and the right eye flicker fusion frequency in Assessment 3 was significantly higher than in Assessments 1 and 2 (*p* < 0.001). The right eye flicker fusion frequency in Assessment 2 was significantly higher than in Assessment 1 (*p* < 0.001). In higher grades, the right eye flicker fusion frequency of the three assessments demonstrated significant differences (F = 3.718, *p* < 0.05), of which only Assessments 1 and 3 of the right eye flicker fusion frequency demonstrated significant differences (*p* = 0.030). The right eye flicker fusion frequency in the third assessment was significantly higher than that in the first.

These ANOVA results thus indicate the following: (1) The horary visual acuity of both eyes was worse in higher grade students than in lower grade students; and (2) in both higher and lower grade students, horary visual acuity decreased with each timepoint (i.e., longer use of thee E-learning environment), with the horary visual acuity changes being more significant in lower-grade students.

Thus, the two-factor mixed-design ANOVA provided results that are close to those of single-factor ANOVA. However, the results of the two-factor mixed-design ANOVA for vision function and depth perception are not detailed here.

### 5.2. Relationship between Duration of Use and Visual Acuity in E-learning Environments

All students were ranked according to the time they spent using E-learning environments to exclude missing values. Finally, 561 valid samples were obtained. All respondents used E-learning environments in and after class for 0–30 h/week (average = 7.5 h/week).

Analysis of covariance (ANCOVA) was performed to explore E-learning environment use duration (in and after class) with elementary and middle school students’ visual acuity. Here, the weekly E-learning environment use duration was the independent variable, and students’ left eye visual acuity values in Assessments 2 and 1 were the dependent variable and covariate, respectively. First, the homogeneity test of the regression coefficient within the group was employed. The interaction between the independent variable and covariate was F (1, 479) = 0.358, *p* > 0.05, which did not reach a significant level, and thus indicated a linear relationship between the covariate and the dependent variable in each group; consequently, the ANCOVA could be continued. To correctly estimate the effects of independent variables and covariance, the interaction terms were removed and reanalyzed. Levene’s variance homogeneity test yielded nonsignificant results (F (1, 481) = 0.016, *p* > 0.05), indicating no significant differences in the dispersion of the two samples. The covariance reached a significant level (F (1, 480) = 905.533, *p* < 0.01) and satisfied the conditions for a linear relationship. The test for between-group effects demonstrated significant results (F (1, 480) = 18.940, *p* < 0.01), indicating that the time spent using the E-learning environment significantly affected students’ left eye visual acuity. Table 7 summarizes the ANCOVA results for left eye visual acuity.

Similarly, the weekly duration of E-learning environment use by the elementary and middle school students was considered as the independent variable, and students’ right eye visual acuity in Assessments 2 and 1 as the dependent variable and covariate, respectively. The results revealed that E-learning environment use duration significantly affected students’ right eye visual acuity (F (1, 482) = 4.130, *p* < 0.05). The right eye visual acuity ANCOVA results are summarized in Table 8.

Therefore, in general, E-learning environment use duration is significantly correlated with student vision.

To further assess whether E-learning environment use duration (in and after class) has a positive or negative impact on the vision of elementary and middle school students, the samples were divided into two groups of equal numbers. Group 2 contained 281 students with heavy use of E-learning environments (i.e., E-learning environment use of 14–30 periods per week). The grade distribution of students in the two groups was basically the same. Compared with the mean value after covariance correction, light E-learning environment use (left eye = 4.91 and right eye = 4.89) led to significantly better visual acuity than heavy use (left eye = 4.89 and right eye = 4.88; F (1, 480) = 18.940, *p* < 0.01 and F (1, 482) = 4.130, *p* < 0.05, respectively).

The difference in the test results of the three test data between the experimental class (G4) and the control class in Grade 4, as mentioned above, shows that the measurement data in a 1.5-year E-learning environment use cycle demonstrated that the current E-learning environment use duration and frequency (10.12 h/week; approximately 6.75 h on average) did not significantly affect vision in the students in higher grades of elementary school (Grades 4 and 5). This may be related to the particularity of the upper grade of elementary school, noted in Section 4.1.3: The vision of the students remained stable in G4, indicating that the higher grades of elementary school are exceptions.

Because of the lack of appropriate data and studies thus far, the relationship of E-learning environment use duration with visual field, depth perception, and horary visual acuity could not be analyzed further.

## 6. Conclusions

### 6.1. Visual Acuity Features

(1) With respect to students with poor visual acuity educated under the E-learning environment, the poor visual acuity prevalence of lower grade elementary school students (Grades 2–4) was lower than the values (45.71%) in the 2014 China Student Physique and Health Research Report, published by China’s Ministry of Education, General Administration of Sport, and other relevant departments. In higher grade elementary school students (Grades 5 and 6), poor visual acuity prevalence was higher than that in the lower grade elementary school students but lower than that in the aforementioned 2014 national survey (74.4%). However, the visual impairment prevalence in Grade 3 students was higher than in the results. The overall situation is worrying;
(2) With the movement to higher grades, the overall prevalence of poor visual acuity gradually increased in students. Over 2 years, the prevalence of poor visual acuity in the elementary and middle school students increased by an average of 12.24%—with the prevalence being the highest for students in Grade 1 of middle school (14.66%). This result is consistent with a previous result [37];
(3) The horizontal comparison of differences in the visual acuity of students in different grades at the same timepoint (including three assessment timepoints) demonstrated that the visual acuity of students in higher grades was poorer, and the visual acuity of the middle school students was significantly lower than that of the Grades 2–4 students. With the passing of each semester, the students’ visual acuity decreased significantly, consistent with real-life observations;
(4) On the basis of the vertical changes in the visual acuity of students of the same grade at different timepoints, during the 2-year period of E-learning environment use, the visual acuity in G2 improved first (i.e., after use for two semesters) and then decreased (after use for three to four semesters); similarly, in G3, visual acuity remained unchanged at first and then decreased, whereas, in G4, no significant change was noted. However, in G7, visual acuity worsened first and then improved, potentially because of the increased sports intervention in Grade 3 of middle school, necessary to fulfil the requirements of the middle school-level vocational examinations. In each Chinese province, the specific intervention programs and intensities differ;
(5) The two-factor mixed-design ANOVA results corroborated the aforementioned conclusions; and
(6) For all elementary and middle school students, E-learning environment use duration significantly affected left and right eye visual acuity, except for that of Grade 4 students in elementary school. According to the published literature, in addition to in- and after-class use of E-learning environments provided by the school, the main reasons for the decline in students’ visual acuity may include the use of other related electronic products, a high schoolwork burden [38], high study pressure [39], a reduction in exercise, lighting conditions, and genetic factors.

### 6.2. Visual Field Features

(1) The horizontal comparison of differences in the visual field of students in different grades at the same timepoint (including three assessment timepoints) demonstrated that students in higher grades had a better visual field than students in lower grades;
(2) The vertical changes in the visual field of the students of the same grade at different assessment timepoints indicated that, over 2 years, the upper, lower, inner, and outer visuals fields of both eyes of the students gradually changed with age. This result is consistent with a previous result [22]; and
(3) The differences in the results of the three assessments’ data between G4 and Control demonstrated that when they used E-learning environments for a short period (about two semesters), the visual field of older elementary school students narrowed or centralized, whereas long-term use (three semesters, i.e., ≥ 1.5 years) led to their visual field deviating toward the lower left (this may be related to the “phubbing phenomenon”).

### 6.3. Depth Perception Features

(1) The horizontal comparison of differences in the depth perception of students in different grades at the same timepoint (including the three assessment timepoints) demonstrated that, on the whole, the depth perception of senior students was worse;
(2) On the basis of the vertical changes in the visual acuity of students of the same grade at different timepoints, in the short term (two semesters, i.e., within 1 year), the depth perception of students in lower grades in elementary school (G2 and G3) improved significantly, but it worsened in the long term (three or more semesters, i.e., 1.5–2 years); by contrast, the students in middle school demonstrated the opposite trend, and G4 demonstrated no significant changes; and
(3) The absence of differences in the data results of the three assessments between G4 and Control indicated that, at a certain use frequency, E-learning environment use was not the main factor influencing the depth perception of older elementary school students.

### 6.4. Horary Visual Acuity Features

(1) The horizontal comparison of differences in the horary visual acuity of students in different grades at the same timepoint (including the three assessment timepoints) demonstrated that the higher the grade a student was in, the higher the horary visual acuity;
(2) On the basis of the vertical changes in the horary visual acuity of students of the same grade at different timepoints, in the short term (about two semesters), the horary visual acuity improved in all elementary school students; however, in the long term (four semesters and above), the horary visual acuity of lower grade elementary school students (G2 and G3) improved but that of higher grade elementary school students (G4) worsened. In middle school students, horary visual acuity remained unchanged in the left eye but improved in the right eye; and
(3) The differences in the data results of the three assessments between G4 and Control demonstrated that when they used E-learning environments for a short period (two semesters), higher grade elementary school students did not demonstrate significant changes in their horary visual acuity, whereas long-term use (three semesters, i.e., ≥ 1.5 years) led to a significant improvement in right eye horary visual acuity.

## 7. Implications

In conclusion, the following are problems that require the attention of students, parents, schools, governments, and society: Firstly, the proportion of students with poor vision continues to increase as they increase in grade. Secondly, E-learning environment use can significantly affect elementary and middle school students’ vision. Thirdly, for elementary school students in Grades 4 and 5, the current E-learning environment use duration and frequency (mainly referring to the use of E-learning equipment provided by the school in and out of class) is not the main factor that affects their vision and depth perception; it can even improve the time horary visual acuity of students’ right eyes. However, long-term use can lead to gradual changes, such as the deterioration of right eye horary visual acuity, in higher grade elementary school students. However, the visual field can become narrow, with deviation to the lower left (this may be related to the “phubbing phenomenon”). Fourth, in students, the main causes of vision loss, in addition to the E-learning environment use after class, may be the increased use of other related electronic products (such as smartphones, televisions, and gaming consoles), increased schoolwork burden and study pressure, and decreased exercise.

## Figures and Tables

**Table 1:** Single-factor ANOVA results for Assessment 3 left eye visual acuity data.

Source of Variation		SS	Df	MS	F	Post-Hoc Comparisons
Between groups		2.422	1.454	1.666	47.322 **	3 < 1 < 2
In groups	Between subjects	15.116	85	0.178		
	Residual	4.351	123.601	0.035		
Sum		21.889	210.055			

Note: ** *p* < 0.01; SS = Sum of Squares, Df = Degrees of Freedom, MS = Mean Square.

**Table 2:** Visual field changes in the left and right eyes in G2 in all three assessments.

Visual Field Test	Left Upper Visual Field	Left Interal Visual Field	Left Lower Visual Field	Left Lateral Visual Field	Right Upper Visual Field	Right Interal Visual Field	Right Lower Visual Field	Right Lateral Visual Field
First	33.90	42.97	44.97	52.51	34.42	44.45	45.24	51.54
Second	44.34	53.57	60.21	68.18	43.54	55.49	59.81	68.88
Third	47.08	57.96	60.01	66.07	47.15	57.63	59.80	68.18

**Table 3:** Changes in the average apparent depth in all students over 2 years.

Grade	Average	Grade	Average	Grade	Average	Grade	Average
G2 test1	1.12	G3 test1	1.16	G4 test1	1.54	G7 test1	1.16
G2 test2	0.45	G3 test2	0.64	G4 test2	1.38	G7 test2	2.22
G2 test3	0.94	G3 test3	0.91	G4 test3	0.90	G7 test3	1.25

**Table 4:** Comparison of the flicker fusion frequency in three assessments in all students.

Test	Left	Right	Test	Left	Right	Test	Left	Right	Test	Left	Right
G2 test1	30.1	29.7	G3 test1	33.8	31.2	G4 test1	34.3	33.4	G7 test1	44.0	45.1
G2 test2	44.1	43.8	G3 test2	43.9	44.1	G4 test2	43.3	44.2	G7 test2	46.5	46.3
G2 test3	49.1	48.5	G3 test3	47.3	46.9	G4 test3	42.2	43.8	G7 test3	46.0	51.1

**Table 5:** Two-factor mixed-design ANOVA results for left eye visual acuity.

Source of Variation	SS	Df	MS	F	*p*
Grade (independent factor)	6.977	1	6.977	32.858	0.000
Measuring time (correlation factor)	0.129	1.715	0.075	2.461	0.095
Grade × measuring time	1.190	1.715	0.694	22.762	0.000
In groups	110.353	1132.196			
Between subjects	88.550	417	0.212		
Residual	21.803	715.196	0.030		
Sum	118.649	418			

**Table 6:** Two-factor mixed-design ANOVA results for right eye visual acuity.

Source of Variation	SS	Df	MS	F	*p*
Grade (independent factor)	12.996	1	12.996	65.185	0.000
Measuring time (correlation factor)	0.081	1.765	0.046	1.563	0.212
Grade × measuring time	1.590	1.765	0.901	30.862	0.000
In groups	102.614	1130.769			
Between subjects	81.540	409	0.199		
Residual	21.074	721.769	0.029		
Sum	117.281	410			

**Table 7:** Left eye visual acuity ANCOVA results.

Source of Variation	SS	Df	MS	F
Covariance	33.448	1	33.448	905.533
Between subjects	0.700	1	0.700	18.940
In groups (error)	17.730	480	0.037	
Sum	11,638.910	483		

**Table 8:** Right eye visual acuity ANCOVA results.

Source of Variation	SS	Df	MS	F
Covariance	33.319	1	33.319	888.585
Between subjects	0.155	1	0.155	4.130
In groups (error)	18.074	482	0.037	
Sum	11,621.060	485		
